# Genetic variants of m^6^A modification genes are associated with survival of HBV‐related hepatocellular carcinoma

**DOI:** 10.1111/jcmm.18517

**Published:** 2024-08-20

**Authors:** Shuyan Liu, Jianxu Li, Moqin Qiu, Yingchun Liu, Qiuping Wen, Qiuling Lin, Yanji Jiang, Zihan Zhou, Xiumei Liang, Xiaoxia Wei, Hongping Yu, Peiqin Chen

**Affiliations:** ^1^ Guangxi Medical University Nanning Guangxi China; ^2^ Department of Radiation Oncology Guangxi Medical University Cancer Hospital Nanning Guangxi China; ^3^ Department of Respiratory Oncology Guangxi Medical University Cancer Hospital Nanning Guangxi China; ^4^ Department of Experimental Research Guangxi Medical University Cancer Hospital Nanning Guangxi China; ^5^ Department of Clinical Research Guangxi Medical University Cancer Hospital Nanning Guangxi China; ^6^ Scientific Research Department Guangxi Medical University Cancer Hospital Nanning Guangxi China; ^7^ Department of Cancer Prevention and Control Guangxi Medical University Cancer Hospital Nanning Guangxi China; ^8^ Department of Disease Process Management Guangxi Medical University Cancer Hospital Nanning Guangxi China; ^9^ Key Laboratory of Early Prevention and Treatment for Regional High Frequency Tumor (Guangxi Medical University) Ministry of Education Nanning Guangxi China; ^10^ Editorial Department of Chinese Journal of Oncology Prevention and Treatment Guangxi Medical University Cancer Hospital Nanning Guangxi China

**Keywords:** genetic variants, hepatitis B virus, hepatocellular carcinoma, m^6^A, survival

## Abstract

N^6^‐methyladenosine (m^6^A) is a dynamic and reversible modification process involving in a series of important biological and pathophysiological processes, including the progression of cancers. Herein, we aimed to assess the relationships of genetic variants in m6A modification genes with the survival of hepatitis B virus ‐related hepatocellular carcinoma (HBV‐HCC). We performed a two‐stage survival analysis to investigate the associations of 4425 single nucleotide polymorphisms (SNPs) in 36 m^6^A modification genes with the overall survival (OS) of HBV‐HCC patients. Then, the identified SNPs were further used to functionally annotate. We identified that *METTL3* rs1263790 (A > G) and *ADARB1* rs57884102 (C > T) were significantly associated with the HBV‐HCC OS (hazard ratios [HR] = 0.68, 95% confidence interval [CI] = 0.52–0.89, *p* = 0.004; and HR = 1.70, 95% CI = 1.33–2.18, *p* < 0.001, respectively). Combined analysis revealed that patients carrying more risk genotypes of two variants had a progressively poorer OS. Moreover, the expression quantitative trait loci (eQTL) analysis indicated that rs1263790 G allele decreased mRNA expression levels of *METTL3* in 483 cell‐cultured fibroblasts samples. And we found the mRNA expression levels of *METTL3* and *ADARB1* in HCC tissues were higher than in normal tissues, and the higher *METTL3* and the lower *ADARB1* were associated with poorer HCC OS. Our results demonstrated that two novel genetic variants (*METTL3* rs1263790 and *ADARB1* rs57884102) may be potential prognostic markers for HBV‐HCC, but these results need larger different ethnic cohorts and functional experiments to validate in the future.

## 1 INTRODUCTION

Globally, liver cancer is the sixth most prevalent cancer and the third cancer‐related cause of death in 2020, with approximately 906,000 new cases diagnosed, and half of all cases occurred in China.^1^ Hepatocellular carcinoma (HCC) is the primary histologic type of liver cancer, accounting for approximately 90% of liver cancer patients.^2^ Hepatitis B virus (HBV) causes 84.4% of HCC in China, and actively promotes the progression of HCC.^3^ Although the treatment of HCC has advanced over the past decades, the 5‐year overall survival (OS) rate of HCC remains dismal with only 12.1% in China between 2012 and 2015,^4^ even for patients with early‐stage HCC who undergo hepatectomy only have a survival rate about 70%.^5^ Therefore, the discovery of applicable biomarkers would help improve prognosis of patients with HCC. Some clinical characteristics, such as alpha fetoprotein (AFP), microvascular invasion, Barcelona Clinic Liver Cancer (BCLC) stage and treatment methods, are used to predict HCC survival.^6^ At present, genome‐wide association studies (GWAS) have successfully confirmed that single‐nucleotide polymorphisms (SNPs), the most common type of genetic variants, have been reported to associate with the prognosis of patients with HCC and may serve as the biomarker signature of clinical prognosis.^7^

N^6^‐methyladenosine (m^6^A), the most prevalent type of mRNA modification in eukaryotes, is a phenomenon that affects the 6th nitrogen atom of RNA molecule adenine, primarily in the coding sequence section (CDS) and the 3′‐untranslated region (3′‐UTR) of mRNA. M^6^A methylation is affected by different types of regulators such as methyltransferase (writers), demethylase (erasers) and related binding proteins (readers), and it is a dynamic and reversible alteration procedure that controls RNA metabolism activities, such as alternative splicing, export, stability and translation.^8^, ^9^ Studies have suggested that the important biological and pathophysiological processes, including sperm development, embryonic growth, reprogramming, proliferation, differentiation and apoptosis, have been linked to m^6^A methylation alteration.^10^, ^11^, ^12^, ^13^ Recent reports have showed that disturbed activities of ‘writers’, ‘erasers’ or ‘readers’ often result in abnormal m^6^A methylation modification, thus regulating the development and progression of cancers, including HCC.^14^, ^15^, ^16^ Presently, a number of studies have demonstrated the relationships of genetic variants of m^6^A modification genes with cancers.^17^, ^18^, ^19^, ^20^

Given the important role of m^6^A methylation modification in HCC, we hypothesized that genetic variants of m^6^A modification genes may be associated with HBV‐HCC patient's survival. In the present study, we conducted a two‐stage survival analysis to test this hypothesis in a Chinese population of patients with HBV‐HCC after hepatectomy.

## 2 MATERIALS AND METHODS

### 2.1 Study populations

This study recruited 866 HCC patients from Guangxi Medical University Cancer Hospital between June 2007 and December 2017.^21^, ^22^, ^23^ All participants were diagnosed with histologically confirmed HCC and all cases in the study were tested seropositive for HBV surface antigen (HBsAg). The OS was based on the time from hepatectomy to the last follow‐up or death. The deadline for follow‐up was March 31, 2020, with a success rate of 84.5%. The 866 HBV‐related HCC (HBV‐HCC) patients were randomly split (1:1) into discovery and replication groups. Before participating, each participant signed a written informed consent in this study. Moreover, the study protocol was approved by the Institutional Review Board of Guangxi Medical University Cancer Hospital (LW2023106).

### 2.2 Genes and SNPs selection

The 36 m^6^A modification genes were selected from three published literatures,^24^, ^25^, ^26^ all of which were located on the autosomal chromosomes (Table S1). Gene genotyping was performed using the Illumina Infinium Global Screening Assay (GSA, Illumina, San Diego). SNPs within 2 kb upstream and downstream of 36 m^6^A modification genes were extracted for further analysis. SNPs were retained with the following quality control criteria: (1) a genotyping call rate ≥ 95%; (2) the minor allele frequency (MAF) ≥ 5%; and (4) Hardy–Weinberg equilibrium (HWE) *p* ≥ 10^−6^.

### 2.3 RNA sequencing

We further used RNA sequencing data of 100 paired HCC tissues and normal adjacent tissues collected from Guangxi Medical University Cancer Hospital after consent to analyse the differences in mRNA expression levels.

### 2.4 Functional prediction

We used SNPinfo (https://snpinfo.niehs.nih.gov/snpinfo/snpfunc.html), RegulomeDB (http://regulomedb.org/) and Haploreg 4.1 (http://archive.broadinstitute.org/mammals/ haploreg) to predict potential functions for SNPs. The expression quantitative trait loci (eQTL) analysis of the identified SNPs and mRNA expression levels of the corresponding genes used the data from the GTEx database (https://www.gtexportal.org/home/) and 1000 Genomes Project. Then, we compared the gene expression between HCC tissues and adjacent normal tissue via using the Cancer Genome Atlas (TCGA) database (http://ualcan.path.uab.edu/analysis.html), and assessed the associations of gene expression with OS of HCC patients from the Kaplan–Meier Plotter website (http://kmplot.com/analysis/index.php?p=service).

### 2.5 Statistical analysis

We first performed multivariable Cox proportional hazards regression models to evaluate the associations between SNPs and OS of HBV‐HCC patients in an additive genetic model by the GenABEL and gap packages of R software (version 3.1.3), with adjustment for age, sex, smoking status, smoking status, drinking status, cirrhosis, cancer embolus, BCLC stage and AFP level in the discovery and replication datasets. Considering that many SNPs under research were in high linkage disequilibrium (LD) attribute to imputations, the Bayesian false‐discovery probability (BFDP) with a cut‐off value of 0.8 was recommended to decrease the probability of false‐positive findings for multiple comparisons.^27^ After that, we used a multivariable stepwise Cox model with adjustment for clinical variables to verify independent SNPs associated with the OS of HBV‐HCC. Meanwhile, we combined the risk genotypes of identified SNPs to calculate their cumulative effects. In the stratified analysis, we calculated possible interactions among subgroups of each clinical variables.

Additionally, we conducted bootstrapping with 1000 replicates and calculated the hazard ratios (HR) to assess whether significant SNPs were caused by chance randomization in an additive genetic model by using the multivariable Cox regression analysis. Kaplan–Meier survival curves and log‐rank tests were used to visually evaluate the effects of SNPs on the cumulative probability of OS. Haploview v4.2 was applied to construct the Manhattan plots. LocusZoom (http://locuszoom.org/genform.php?type=yourdata) was carried out to generate the regional association plots.

All statistical computation was performed by PLINK (version 1.90) and R software (version 4.0.0), the result was estimated significant when *p* < 0.05 by two‐sided test.

## 3 RESULTS

### 3.1 Associations of SNPs in the m^6^A modification genes with HBV‐HCC OS

**FIGURE 1:**
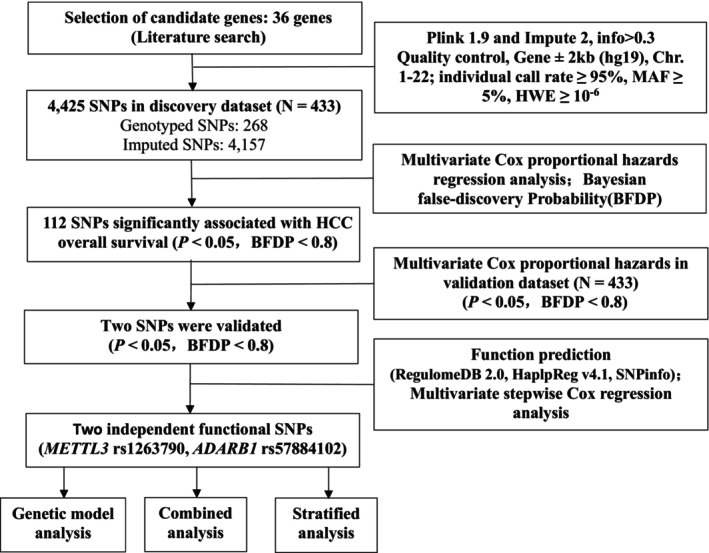
Flow chart of the analysis process.

The overall workflow chart of this study is shown in Figure 1. The associations of demographics and clinical characteristics with OS in 866 HBV‐HCC patients have been described in previous published study (Table S2).^21^, ^22^, ^23^ In the discovery dataset, we identified that 112 SNPs of m^6^A modification genes were significantly associated with HBV‐HCC OS after multiple testing correction (BFDP ≤0.80) in an additive genetic model. Then we further replicated these SNPs in the replication dataset. As a result, we found that rs1263790 in methyltransferase like 3 (*METTL3*) and rs57884102 in adenosine deaminase RNA‐specific B1 (*ADARB1*) were associated with OS of HBV‐HCC patients (HR = 0.68, 95% CI = 0.52–0.89, *p* = 0.004 for rs1263790 and HR = 1.70, 95% CI = 1.33–2.18, *p* < 0.001 for rs57884102, in combined dataset, Table 1). The Manhattan plot of associations between these SNPs and HBV‐HCC OS is shown in Figure S1.

**TABLE 1:** Associations of two significant SNPs with OS of patients with HBV‐HCC.

SNP	Gene	Chr	Position	Discovery dataset			Repilcation dataset			Combined dataset		
				MAF	HR (95% CI)^a^	*p* ^a^	MAF	HR (95% CI)^a^	*p* ^a^	MAF	HR (95% CI)^a^	*p* ^a^
rs1263790 A > G	*METTL3*	14	21,968,619	0.075	0.64 (0.42–0.97)	0.038	0.096	0.69 (0.49–0.98)	0.032	0.085	0.68 (0.52–0.89)	0.004
rs57884102 C > T	*ADARB1*	21	46,556,554	0.080	1.85 (1.32–2.60)	<0.001	0.058	1.63 (1.12–2.36)	0.008	0.069	1.70 (1.33–2.18)	<0.001

Abbreviation: AFP, Alpha‐Fetoprotein; BCLC, Barcelona Clinic Liver Cancer Classification; CI, Confidence Interval; HBV, Hepatitis B Virus; HCC, Hepatocellular Carcinoma; HR, Hazards Ratio; MAF, Minor Allele Frequency; OS, Overall Survival.

^a^ Obtained from the single‐locus multivariable Cox regression analysis with adjustment for age, sex, smoking status, drinking status, cirrhosis, cancer embolus, BCLC stage and AFP level.

### 3.2 Identification of independent SNPs associated with HBV‐HCC OS

We further performed functional prediction of the two identified SNPs by using SNPinfo, RegulomeDB and Haploreg (Table 2). We found that these two identified SNPs both located in the intron regions and had certain functions. In RegulomeDB, *METTL3* rs1263790 and *ADARB1* rs57884102 had both scores of 5, indicating the potential transcriptional factor binding or DNase peak. Functional annotation of two SNPs in HaploReg demonstrated that *METTL3* rs1263790 and *ADARB1* rs57884102 both overlapped with the enhancer; *METTL3* rs1263790 potentially disrupted two motifs, including Bbx and Sox and affected the mRNA expression; similarly, *ADARB1* rs57884102 might disrupt 10 motifs. However, no information with functional annotations was found in SNPinfo. Then, we also performed the multivariable stepwise Cox regression analysis and found that these two identified SNPs (*METTL3* rs1263790 A > G and *ADARB1* rs57884102 C > T) were independently associated with the OS of HBV‐HCC patients in the combined dataset (HR = 0.68, 95% CI = 0.52–0.88, *p* = 0.004 for rs1263790 and HR = 1.72, 95% CI = 1.34–2.20, *p* < 0.001 for rs57884102, in an additive genetic model, Table 3).

**TABLE 2:** Functional prediction of two significant SNPs.

SNP	gene	SNPinfo^a^	RegDB 2.0^b^ score	Haploreg v4.1^c^				
				Promoter histone marks	Enhancer histone marks	DNAse	Motifs changed	Location
rs1263790	METTL3	—	5	—	ESC, BLD	IPSC, BLD	Bbx, Sox	Intronic
rs57884102	ADARB1	—	5	—	11 tissues	MUS	10 altered motifs	Intronic

^a^ http://snpinfo.niehs.nih.gov/snpinfo/snpfunc.htm.

^b^ http://www.regulomedb.org/index.

^c^ http://archive.broadinstitute.org/mammals/haploreg/haploreg.php.

**TABLE 3:** Independent predictors of OS of patients with HBV‐HCC in stepwise multivariable Cox regression analysis of selected variables by the combined datase.

Characteristics	Category	Frequency	HR (95% CI)^a^	*p* ^a^
Age (year)	≤47/>47	434/432	0.77 (0.63–0.93)	0.008
AFP(ng/mL)	≤400/>400	522/344	1.31 (1.07–1.61)	0.008
Cancer embolus	No/Yes	636/230	1.77 (1.40–2.24)	<0.001
BCLC stage	0/A stage/B/C stage	427/439	2.04 (1.61–2.59)	<0.001
*METTL3* rs1263790^b^	AA/AG/GG	369/63/1	0.68 (0.52–0.88)	0.004
*ADARB1* rs57884102^b^	CC/CT/TT	367/63/3	1.72 (1.34–2.20)	<0.001

Abbreviation: AFP, Alpha‐Fetoprotein; BCLC, Barcelona Clinic Liver Cancer Classification; CI, Confidence Interval; HBV, Hepatitis B Virus; HCC, Hepatocellular Carcinoma; HR, Hazards Ratio; OS, Overall survival.

^a^ Obtained from the stepwise multivariable Cox regression analysis with adjustment for age, sex, smoking status, drinking status, cirrhosis, cancer embolus, BCLC stage, AFP level, *METTL3* rs1263790 and *ADARB1* rs57884102.

^b^ All SNPs used an additive genetic model.

**FIGURE 2:**
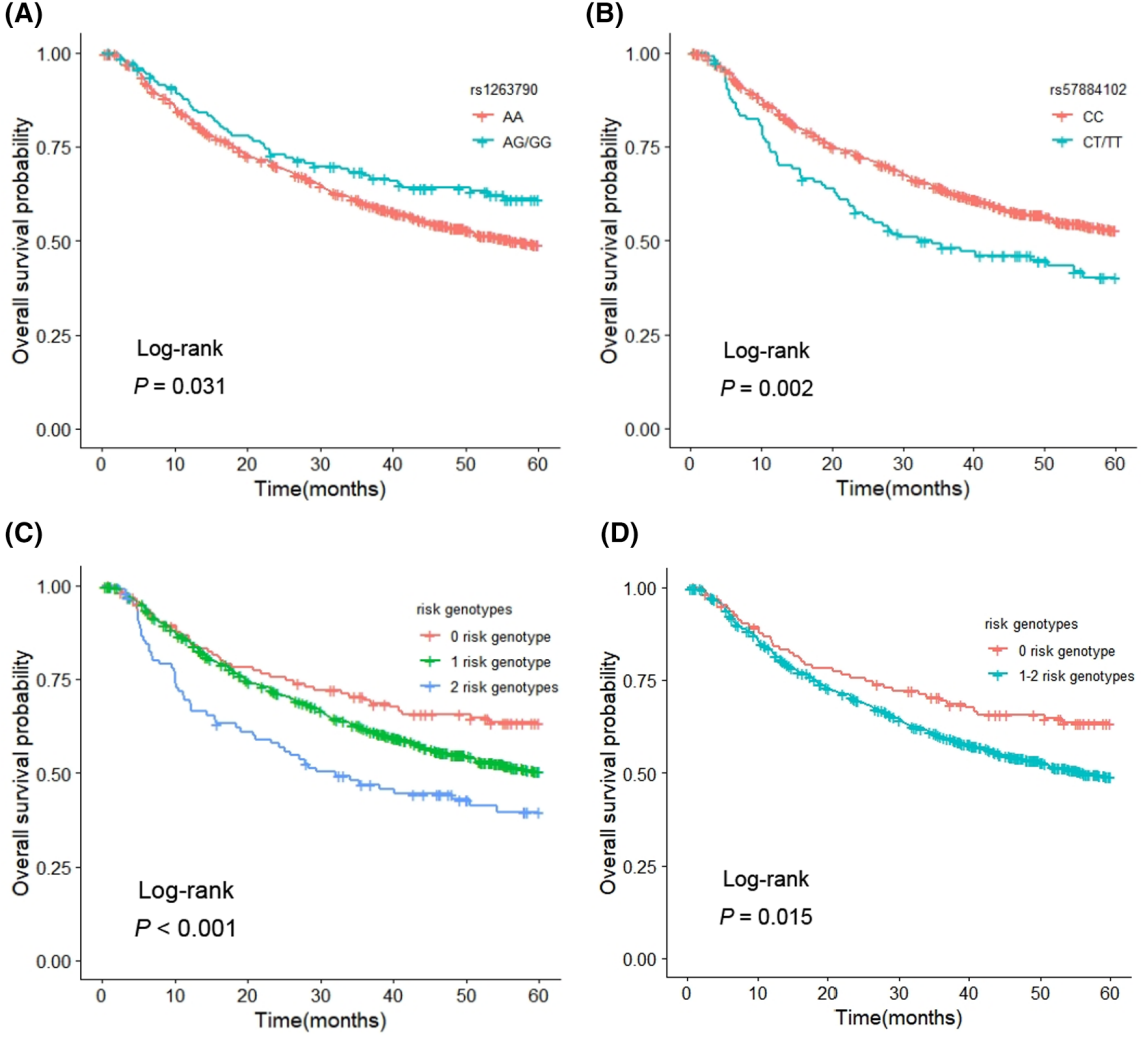
Kaplan–Meier curves for the SNPs and 5‐years OS of HBV‐HCC in combined dataset. Kaplan–Meier curves for associations of *METTL3* rs1263790 (A) and *ADARB1* rs57884102 (B) in the dominant genetic models with 5‐years OS of HBV‐HCC. Kaplan–Meier curves for associations between combined risk genotypes and 5‐years OS of HBV‐HCC (C, D). OS, Overall Survival; HBV, Hepatitis B Virus; HCC, Hepatocellular Carcinoma.

For each of the two independent SNPs, we further accessed their effects on the risk of death in different genetic models. As shown in Table 4, in the additive genetic model, HBV‐HCC patients with the *METTL3* rs1263790 G allele had a favourable OS (*p*_trend_ = 0.004), and the *ADARB1* rs57884102 T allele was a risk factor for HBV‐HCC OS (*p*_trend_ <0.001) in the combined dataset. In the dominant genetic models, compared with the HBV‐HCC patients carrying *METTL3* rs1263790 AA genotype, the patients carrying AG/GG genotypes had a better OS (HR = 0.68, 95% CI = 0.52–0.90, *p* = 0.007). However, the HBV‐HCC patients with *ADARB1* rs57884102 CT/TT genotypes were associated with a poorer OS than those with CC genotype (HR = 1.72, 95% CI = 1.32–2.25, *p <* 0.001). The 1000 replicates bootstrapping showed that the HR values of the rs1263790 and rs57884102 were included in the 95% CI of repeated sampling (Figure S2), indicated that the associations between these two SNPs and the OS of HBV‐HCC patients were reliable. Kaplan–Meier survival curves of these associations of the two SNPs with HCC OS were depicted in Figure 2A,B (Log‐rank *p* = 0.031 for rs1263790, and Log‐rank *p* = 0.002 for rs57884102), as well as the regional association plots (Figure S3).

**TABLE 4:** Associations between the two independent SNPs in the m^6^A modification genes and OS of HBV‐HCC patients.

Genotype	Discovery dataset				Replication dataset				Combined dataset			
	All	Death (%)	HR (95% CI)^a^	*p* ^a^	All	Death (%)	HR (95% CI)^a^	*p* ^a^	All	Death (%)	HR (95% CI)^a^	*p* ^a^
*METTL3* rs1263790 A > G												
AA	369	175 (47.43)	1.00		354	184 (51.98)	1.00		723	359 (49.65)	1.00	
AG	63	25 (39.68)	0.64 (0.42–0.99)	0.045	75	34 (45.33)	0.72 (0.50–1.06)	0.093	138	59 (42.75)	0.70 (0.53–0.93)	0.013
GG	1	0	—	0.993	4	1 (25.00)	0.30 (0.04–2.16)	0.231	5	1 (20.00)	0.26 (0.04–1.84)	0.176
Trend test				0.038				0.040				0.004
AG/GG	64	25 (39.06)	0.64 (0.42–0.98)	0.040	79	35 (44.30)	0.69 (0.48–1.01)	0.054	143	60 (41.96)	0.68 (0.52–0.90)	0.007
*ADARB1* rs57884102 C > T												
CC	367	165 (44.95)	1.00		384	188 (48.96)	1.00		751	353 (47.00)	1.00	
CT	63	32 (50.79)	1.82 (1.22–2.69)	0.003	48	30 (62.50)	1.59 (1.08–2.36)	0.020	111	62 (55.86)	1.67 (1.27–2.20)	<0.001
TT	3	3 (100.00)	3.82 (1.18–12.39)	0.025	1	1 (100.00)	3.72 (0.51–27.36)	0.197	4	4 (100.00)	3.34 (1.23–9.10)	0.018
Trend test				<0.001				0.010				<0.001
CT/TT	66	35 (53.03)	1.90 (1.30–2.78)	<0.001	49	31 (63.27)	1.62 (1.10–2.39)	0.014	115	66 (57.39)	1.72 (1.32–2.25)	<0.001

Abbreviation: AFP, Alpha‐Fetoprotein; BCLC, Barcelona Clinic Liver Cancer Classification; CI, Confidence Interval; HBV, Hepatitis B Virus; HCC, Hepatocellular Carcinoma; HR, Hazards Ratio; OS, Overall Survival.

^a^ Obtained from multivariable Cox regression analysis with adjustment for age, sex, smoking status, drinking status, cirrhosis, cancer embolus, BCLC stage and AFP level.

### 3.3 Combined effects of the two independent SNPs in the combined dataset

To assess the joint effect of the two independent SNPs on HBV‐HCC OS, we combined the risk genotypes of *METTL3* rs1263790 AA genotype and *ADARB1* rs57884102 CT/TT genotypes into a genetic score as the number of combined risk genotypes, which classified all HCC patients into three groups (0, 1 and 2 risk genotypes). The trend test indicated that an increased number of risk genotypes was associated with an increased risk of death (*p*_trend_ < 0.001) after adjustment for other variables in the combined dataset (Table 5). After dichotomizing the genetic score, all the patients were regrouped into low‐risk group (0 risk genotype) and high‐risk group (1–2 risk genotypes). Compared with low‐risk group, we observed that the HBV‐HCC patients in the high‐risk group were associated with a significantly poorer OS (HR = 1.61, 95% CI = 1.19–2.18, *p* = 0.002; Table 5). The Kaplan–Meier survival curves showed the similar to the results of Cox regression analysis (Log‐rank *p* < 0.05, Figure 2C,D).

**TABLE 5:** Associations between the combined risk genotypes and OS of HBV‐HCC patients.

Risk genotypes^a^	Frequency		Univariable analysis		Multivariable analysis^b^	
	All	Death (%)	HR (95% CI)	*p*	HR (95% CI)	*p*
0	125	50 (40.00)	1.00		1.00	
1	644	313 (48.60)	1.40 (1.03–1.88)	0.030	1.52 (1.12–2.06)	0.007
2	97	56 (57.73)	1.90 (1.30–2.78)	0.001	2.54 (1.72–3.75)	<0.001
Trend test				<0.001		<0.001
0	125	50 (40.00)	1.00		1.00	
1–2	741	369 (49.80)	1.46 (1.08–1.96)	0.013	1.61 (1.19–2.18)	0.002

Abbreviation: AFP, Alpha‐Fetoprotein; BCLC, Barcelona Clinic Liver Cancer Classification; CI, Confidence Interval; HBV, Hepatitis B Virus; HCC, Hepatocellular Carcinoma; HR, Hazards Ratio; OS, Overall Survival.

^a^ Risk genotypes: *METTL3* rs1263790 AA and *ADARB1* rs57884102 CT/TT.

^b^ Obtained from multivariable Cox regression analysis with adjustment for age, sex, smoking status, drinking status, cirrhosis,cancer embolus, BCLC stage and AFP level.

### 3.4 Stratified analysis for associations of combined risk genotypes with HBV‐HCC OS

To investigate whether the effect of the combined risk genotypes on HBV‐HCC OS was confounded by age, sex, smoking status, smoking status, drinking status, cirrhosis, cancer embolus, BCLC stage and AFP, we performed stratified analysis in the combined dataset. As showed in Table 6, compared with HBV‐HCC patients with 0 risk genotype, those with 1–2 risk genotypes had a significantly poorer OS in the subgroup with age ≤ 47, male, never smoking, never/ever drinking, AFP > 400, cancer embolus and BCLC B/C stage. No interactions between combined risk genotypes and each variable on HBV‐HCC OS were observed among these subgroups.

**TABLE 6:** Stratified analysis of the combined risk genotypes and OS in HBV‐HCC patients.

Characteristics	0 risk genotype			1–2 risk genotypes			Multivariable analysis^a^		*p*_inter_
	All	Death (%)	MST	All	Death (%)	MST	HR (95% CI)	*p*	
Age (year)									
≤47	65	31 (47.69)	85.0	369	202 (54.74)	44.3	1.61 (1.09–2.37)	0.016	0.809
>47	60	19 (31.67)	97.4	372	167 (44.89)	69.5	1.54 (0.95–2.50)	0.078	
Sex									
Female	11	0	—	95	42 (44.21)	64.3	—	0.997	0.989
Male	114	50 (43.86)	97.4	646	327 (50.62)	55.4	1.50 (1.11–2.03)	0.009	
Smoking status									
Never	81	30 (37.04)	115.3	464	238 (51.29)	56.3	1.75 (1.19–2.58)	0.005	0.34
Ever	44	20 (45.45)	97.4	277	131 (47.29)	55.4	1.36 (0.83–2.21)	0.221	
Drinking status									
Never	94	37 (39.36)	97.4	520	255 (49.04)	61.3	1.57 (1.11–2.23)	0.012	0.942
Ever	31	13 (41.94)	81.0	221	114 (51.58)	52.0	1.86 (1.02–3.38)	0.043	
AFP (ng/mL)									
≤400	76	32 (42.11)	97.4	446	200 (44.84)	69.7	1.11 (0.76–1.62)	0.601	0.006
>400	49	18 (36.73)	120.3	295	169 (57.29)	31.3	2.44 (1.46–4.06)	<0.001	
Cirrhosis									
No	63	24 (38.10)	97.4	327	160 (48.93)	56.2	1.72 (1.10–2.68)	0.017	0.705
Yes	62	26 (41.94)	85.0	414	209 (50.48)	56.3	1.51 (1.00–2.29)	0.052	
Cancer embolus									
No	92	31 (33.70)	113.9	544	229 (42.10)	82.6	1.54 (1.06–2.25)	0.025	0.63
Yes	33	19 (57.58)	23.1	197	140 (71.07)	23.7	1.63 (1.00–2.67)	0.049	
BCLC stage									
0/A	60	18 (30.00)	115.3	367	128 (34.88)	99.6	1.42 (0.86–2.34)	0.166	0.441
B/C	65	32 (49.23)	50.7	374	241 (64.44)	28.8	1.64 (1.13–2.39)	0.010	

Abbreviation: AFP, Alpha‐Fetoprotein; BCLc, Barcelona Clinic Liver Cancer Classification; Cl, Confidence Interval; HBV, Hepatitis B Virus; HCC, Hepatocellular Carcinoma; HR, Hazards Ratio; Os, Overall Survival.

^a^ Obtained from multivariable Cox regression analysis with adjustment for age, sex, smoking status, drinking status, cirrhosis,cancer embolus, BCLC stage and AFP level.

### 3.5 eQTL analysis and differential mRNA expression analysis

**FIGURE 3:**
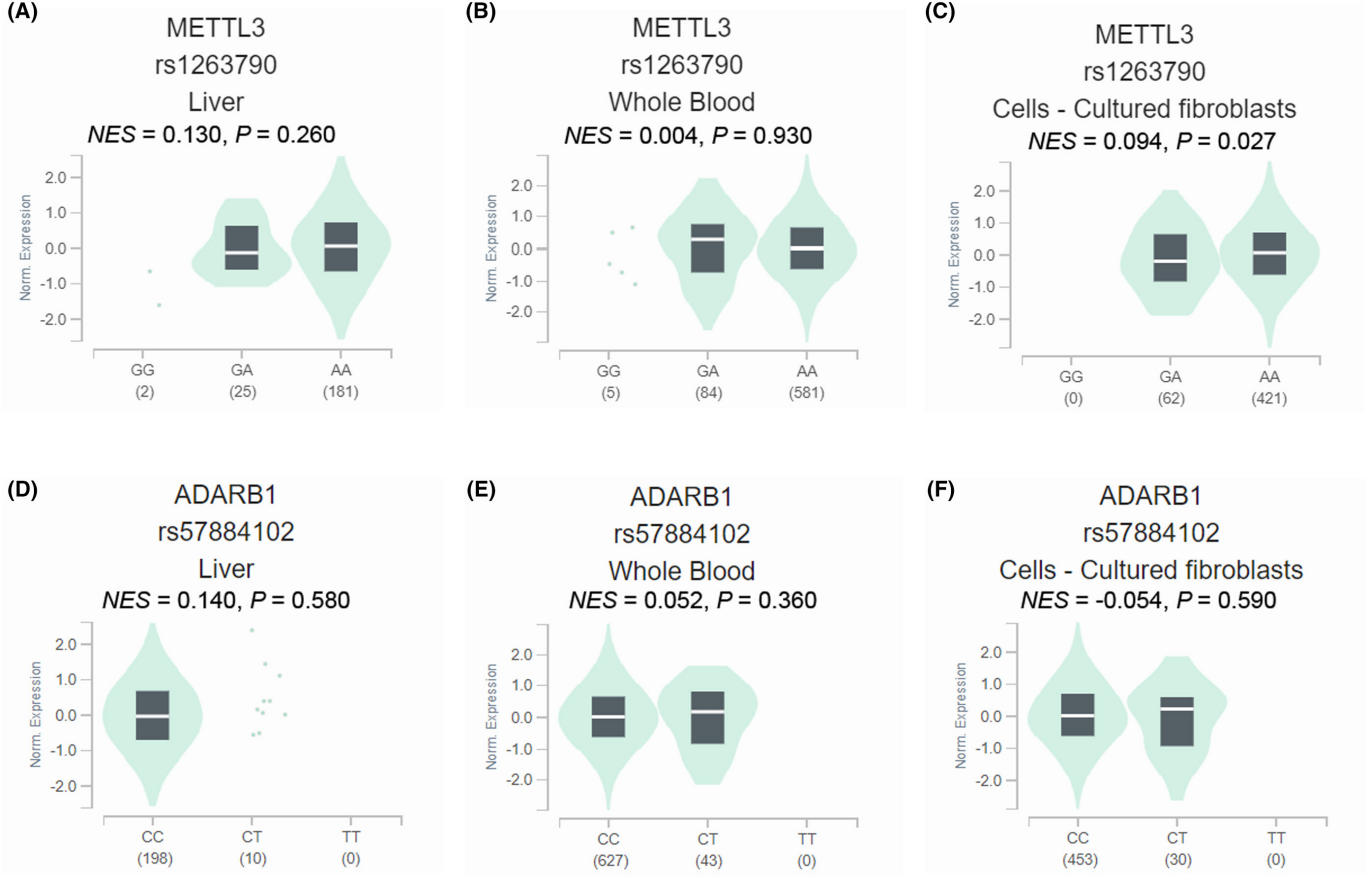
The expression quantitative trait loci (eQTL) analysis for *METTL3* rs1263790 and *ADARB1* rs57884102 in normal liver tissue (A, D), whole blood (B, E) and cell‐cultured fibroblasts samples (C, F) in the GTEx database.

To evaluate correlations between two SNPs (*METTL3* rs1263790 and *ADARB1* rs57884102) and mRNA expression levels of their corresponding genes in the GTEx database, we found no significant correlations of the rs1263790 and mRNA expression levels of *METTL3* in normal liver tissue (*NES* = 0.130, *p* = 0.260, Figure 3A) and whole blood (*NES* = 0.004, *p* = 0.930, Figure 3B), while the rs1263790 A allele was significantly correlated with a higher mRNA expression level of *METTL3* in 483 cell‐cultured fibroblasts samples (*NES* = 0.094, *p* = 0.027, Figure 3C). In addition, the eQTL analysis of rs1263790 in *METTL3* was performed across multiple tissues (Figure S4). However, no significant correlations between rs57884102 and *ADARB1* mRNA expression levels were found in different tissues in the GTEx database (*NES* = 0.140, *p* = 0.580 for normal liver tissue; *NES* = 0.052, *p* = 0.360 for whole blood; *NES* = −0.054, *p* = 0.590 for cell‐cultured fibroblasts samples; Figure 3D–F). The *METTL3* rs1263790, and *ADARB1* rs57884102 were not statistically significant correlated with mRNA expression of their corresponding genes in the Han Chinese in Beijing, China (CHB) populations and Europeans in 1000 Genomes Project (Figure S5, all *p* > 0.05).

**FIGURE 4:**
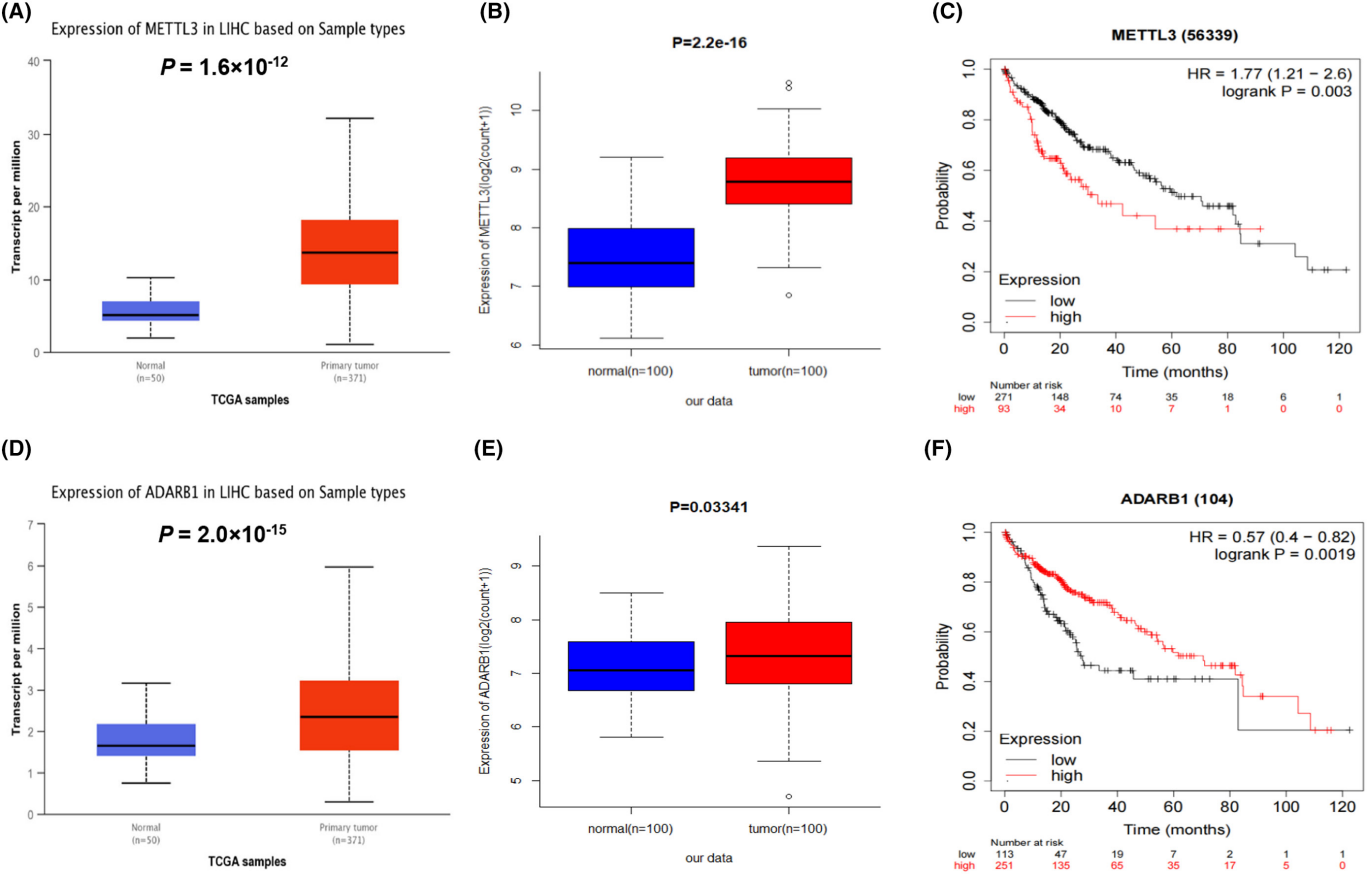
The mRNA expression of genes in HCC tissues and adjacent normal liver tissues and its relationship with OS in HCC patients. The mRNA expression of *METTL3* in HCC tissues and adjacent normal liver tissues in TCGA dataset (A), our dataset (B), and its relationship with OS in HCC (C). patients. The mRNA expression of *ADARB1* in HCC tissues and adjacent normal liver tissues in TCGA dataset (D), our dataset (E) and its relationship with OS in HCC (F). patients. OS, Overall Survival; HCC, Hepatocellular Carcinoma.

Using the TCGA UALCAN database, we evaluated mRNA expression levels in 371 HCC tissues and 50 normal tissues. The results showed that the mRNA expression levels of *METTL3* and *ADARB1* were both significantly higher in HCC tissues than in normal tissues (*p* = 1.6 × 10^−12^ and 2.0 × 10^−15^, respectively; Figure 4A,D), similar results were found in our RNA‐Seq data for the mRNA expression levels of *METTL3* (*p* = 2.2 × 10^−16^, Figure 4B) and *ADARB1* (*p* = 0.03341, Figure 4E). Additionally, we found that HCC patients with lower mRNA expression levels of *METTL3* had a better OS (*p* = 0.003, Figure 4C), while lower mRNA expression levels of *ADARB1* were associated with poorer OS of HCC patients (*p* = 0.0019, Figure 4F).

## 4 DISCUSSION

RNA m^6^A modification plays an essential role in modulating cellular processes, and even affecting the progression of various types of cancer, including HCC.^28^, ^29^ In the present study, we assessed the associations of genetic variants in m^6^A modification genes with HBV‐HCC OS. The result revealed that two potentially functional SNPs (*METTL3* rs1263790 A > G and *ADARB1* rs57884102 C > T) were independently or jointly associated with the OS of HBV‐HCC. The HBV‐HCC patients who carried more risk genotypes of these two SNPs had a progressively poorer OS. Further eQTL analysis showed that rs1263790 G allele might decreased the *METTL3* mRNA expression levels in 483 cell‐cultured fibroblasts samples from GTEx database, but not for *ADARB1* rs57884102. Moreover, the mRNA expression levels of *METTL3* and *ADARB1*were both significantly higher in HCC tissues than in normal tissues from TCGA and our data. And the high mRNA expression levels of *METTL3* were associated with poor OS of HCC patients, while high mRNA expression levels of *ADARB1* were associated with good HCC OS. Thus, we speculated that rs1263790 G allele might contribute to the better OS of HBV‐HCC through decreasing the expression levels of *METTL3*.

*METTL3*, as the only catalytic subunit, locates at chromosome 14q11.2 and contains 580 amino acids that made up of a methyltransferase domain (MTD) and a zinc finger domain (ZFD).^30^ Belonging to the Class I methyltransferase family, METTL3 usually forms stable heterodimer complexes with METTL14 through its methyltransferase activity to mediate m^6^A methylation, regulates a variety of biological processes and plays several functional roles as an oncogene in cancers.^31^, ^32^, ^33^ Recent research demonstrates that METTL3‐mediated m^6^A methylation enhances ANLN mRNA stability via YTHDF1‐dependent manner, thereby promoting HCC bone metastasis.^34^ Above evidence has demonstrated that METTL3 as an m^6^A methyltransferase plays critical roles in progression of HCC. In the present study, we confirmed that the mRNA expression levels of *METTL3* were increased in HCC tissues than in normal tissues from TCGA and our data, and the higher mRNA expression levels of *METTL3* were associated with poorer OS of HCC patients. Additionally, we also found that the rs1263790 G allele of *METTL3* had a protective effect of HBV‐HCC OS, suggesting the genetic variants of *METTL3* might serve as a potential tumour biomarker in HBV‐HCC. Furthermore, *METTL3* rs1263790 overlapped with enhancer regions that might affect transcriptional ability, and rs1263790 G allele might decreased the *METTL3* mRNA expression levels. Considering that METTL3 mediated m^6^A modification of target mRNAs, inhibition of METTL3 can decrease the m^6^A level. Therefore, we believed that the rs1263790 G allele may lead to a reduced transcriptional activity of *METTL3* to decrease the m^6^A modification, leading to improvement of HBV‐HCC survival.

*ADARB1*, one of the ADAR family members, locates at chromosome 21q22.3 and encodes for the ADAR2 protein that played important role in adenosine‐to‐inosine (A‐to‐I) RNA‐editing during tumorigenesis.^35^ Previous studies have revealed that downregulated *ADARB1* contributes to cancer progression with several malignancies, and the lower expression levels of *ADARB1* have been shown to be associated with poor prognosis of patients in different cancers.^35^, ^36^ In the present study, we evaluated the associations between genetic variants of *ADARB1* and HBV‐ HCC OS, and found that the *ADARB1* rs57884102 (C > T) T allele had a significant risk effect on HBV‐HCC OS. It is worth noting that the mRNA expression levels of *ADARB1* in HCC tissues were significantly higher than in normal tissues, yet the higher expression levels of *ADARB1* were associated with better OS of HCC, which may be due to the complexity of the HCC progression process. Nevertheless, there is no direct evidence proving that the *ADARB1* rs57884102 C > T change may regulate the gene expression at the transcription level. Furthermore, we found that the change from rs57884102 C to T might affect enhancer histone mark, but needed to be further validated the biological plausibility of the observed association between the T allele of rs57884102 and HBV‐HCC survival.

To date, no published results have reported the associations between two independent SNPs (rs1263790 and rs57884102) in m^6^A modification genes and HBV‐HCC OS, we reported this association for the first time. However, there are still several limitations in the present study. Firstly, the HBV‐HCC patients were only from Southern China, and the results may not be applied to other ethnic populations; therefore, the results should be further validate in different ethnic HCC patients. Secondly, the sample size of the study population may not be large enough, thus limiting the application of statistics. Finally, we should be further ongoing functional experiments to investigate the intrinsic molecular mechanisms into how the two independent SNPs can affect the m^6^A modification and ultimately impact the survival of HBV‐HCC.

In conclusion, we have identified two potentially functional SNPs (*METTL3* rs1263790 A > G and *ADARB1* rs57884102 C > T) in m^6^A modification genes significantly associated with the survival of HBV‐HCC and may be potential prognostic markers for HBV‐HCC.

## AUTHOR CONTRIBUTIONS

**Shuyan Liu:** Methodology (equal); writing – original draft (equal). **Jianxu Li:** Funding acquisition (equal); writing – review and editing (equal). **Moqin Qiu:** Methodology (equal); project administration (equal). **Yingchun Liu:** Data curation (equal); funding acquisition (equal). **Qiuping Wen:** Formal analysis (equal); investigation (equal); validation (equal). **Qiuling Lin:** Investigation (equal); supervision (equal). **Yanji Jiang:** Investigation (equal); validation (equal). **Zihan Zhou:** Data curation (equal); methodology (equal). **Xiumei Liang:** Formal analysis (equal); supervision (equal). **Xiaoxia Wei:** Conceptualization (equal); methodology (equal). **Hongping Yu:** Funding acquisition (equal); project administration (equal); writing – review and editing (equal). **Peiqin Chen:** Formal analysis (equal); validation (equal).

## CONFLICT OF INTEREST STATEMENT

The authors declare that there is no conflict of interests.

## Supporting information


Appendix S1.

## Data Availability

The data used to support the findings of this study were illustrated in this article. Further inquiries can be directed to the corresponding author.
