# Supplementary material for: Genetic variants of m6A modification genes are associated with survival of HBV‐related hepatocellular carcinoma
Source: J Cell Mol Med. 2024 Aug 20;28(16):e18517. doi: 10.1111/jcmm.18517 (PMC11335057; doi:10.1111/jcmm.18517)
Supplement: Supplementary file 1 — Appendix S1. [file JCMM-28-e18517-s001.docx]

**Genetic variants of m^6^A modification genes are associated with survival of HBV-related hepatocellular carcinoma**

**Supplementary Figures and Tables to:**

**Contents**

**Table S1** The list of 36 m^6^A modification genes.

**Table S2** Associations of demographics and clinical characteristics with OS of HBV-HCC patients.

**Figure S1** Manhattan plot of the association of SNPs and HBV-HCC OS in the combined dataset.

**Figure S2** The distribution histogram of 1000 HR values after grouped by bootstrapping for 1000 times.

**Figure S3** Regional association plots of the *METTL3* rs1263790 and *ADARB1* rs57884102.

**Figure S4** Multi-tissue eQTL analysis of METTL3 rs1263790 from the GTEx database.

**Figure S5** The expression quantitative trait loci (eQTL) analysis for *METTL3* rs1263790 and *ADARB1* rs57884102 from 1000 Genomes Project.

| **Table S1** The list of 36 m^6^A modification genes. | | |
| --- | --- | --- |
| **m^6^A types** | **candidate genes** | **number** |
| Writers | *METTL3 METTL14 METTL16 WTAP RBM15 RBM15B ZC3H13 KIAA1429* | 8 |
| Erasers | *FTO ALKBH5* | 2 |
| Readers or binding proteins | *YTHDF1 YTHDF2 YTHDF3 YTHDC1 YTHDC2 IGF2BP1 IGF2BP2 IGF2BP3 HNRNPC HNRNPA2B1 EIF3A EIF4E LRPPRC ADARB1 DICER1 SND1 DGCR8 DROSHA ADAR PRRC2A SRSF2 EIF3B EIF3C EIF3D EIF3G EIF3I* | 26 |

| Table S2 Associations of demographic and clinical characteristics with OS of HBV-HCC patients | | | | | | | | | | | | | | |
| --- | --- | --- | --- | --- | --- | --- | --- | --- | --- | --- | --- | --- | --- | --- |
| Variables | Discovery dataset | | | |  | Replication dataset | | | |  | Combined dataset | | | |
|  | All | Death (%) | HR (95% CI) a | *P* a |  | All | Death (%) | HR (95% CI) a | *P* a |  | All | Death (%) | HR (95% CI) a | *P* a |
| Age (year) |  |  |  |  |  |  |  |  |  |  |  |  |  |  |
| ≤ 47 | 220 | 117 (53.18) | 1.00 | 0.036 |  | 214 | 116 (54.21) | 1.00 | 0.349 |  | 434 | 233 (53.69) | 1.00 | 0.036 |
| > 47 | 213 | 83 (38.97) | 0.73 (0.55-0.98) |  |  | 219 | 103 (47.03) | 0.88 (0.67-1.15) |  |  | 432 | 186 (43.06) | 0.81 (0.66-0.99) |  |
| Sex |  |  |  |  |  |  |  |  |  |  |  |  |  |  |
| Female | 53 | 22 (41.51) | 1.00 | 0.164 |  | 53 | 20 (37.74) | 1.00 | 0.416 |  | 106 | 42 (39.62) | 1.00 | 0.176 |
| Male | 380 | 178 (46.84) | 1.41 (0.87-2.27) |  |  | 380 | 199 (52.37) | 1.22 (0.76-1.97) |  |  | 760 | 377 (49.61) | 1.26 (0.90-1.76) |  |
| Smoking status | |  |  |  |  |  |  |  |  |  |  |  |  |  |
| No | 254 | 121 (47.64) | 1.00 | 0.769 |  | 291 | 147 (50.52) | 1.00 | 0.509 |  | 545 | 268 (49.17) | 1.00 | 0.475 |
| Yes | 179 | 79 (44.13) | 1.06 (0.73-1.53) |  |  | 142 | 72 (50.70) | 0.89 (0.62-1.27) |  |  | 321 | 151 (47.04) | 0.91 (0.71-1.17) |  |
| Drinking status | |  |  |  |  |  |  |  |  |  |  |  |  |  |
| No | 296 | 137 (46.28) | 1.00 | 0.275 |  | 318 | 155 (48.74) | 1.00 | 0.183 |  | 614 | 292 (47.56) | 1.00 | 0.541 |
| Yes | 137 | 63 (45.99) | 0.81 (0.55-1.19) |  |  | 115 | 64 (55.65) | 1.28 (0.89-1.85) |  |  | 252 | 127 (59.40) | 1.08 (0.84-1.41) |  |
| AFP (ng/mL) | |  |  |  |  |  |  |  |  |  |  |  |  |  |
| ≤ 400 | 258 | 103 (39.92) | 1.00 | 0.004 |  | 264 | 129 (48.86) | 1.00 | 0.581 |  | 522 | 232 (44.44) | 1.00 | 0.015 |
| > 400 | 175 | 97 (55.43) | 1.56 (1.16-2.11) |  |  | 169 | 90 (53.25) | 1.08 (0.82-1.43) |  |  | 344 | 187 (54.36) | 1.29 (1.05-1.57) |  |
| Cirrhosis | |  |  |  |  |  |  |  |  |  |  |  |  |  |
| No | 208 | 96 (46.15) | 1.00 | 0.829 |  | 182 | 88 (48.35) | 1.00 | 0.614 |  | 390 | 184 (47.18) | 1.00 | 0.702 |
| Yes | 225 | 104 (46.22) | 0.97 (0.73-1.29) |  |  | 251 | 131 (52.19) | 1.07 (0.82-1.41) |  |  | 476 | 235 (49.37) | 1.04 (0.85-1.26) |  |
| Cancer embolus | |  |  |  |  |  |  |  |  |  |  |  |  |  |
| No | 319 | 122 (38.24) | 1.00 | < 0.001 |  | 317 | 138 (43.53) | 1.00 | < 0.001 |  | 636 | 260 (40.88) | 1.00 | < 0.001 |
| Yes | 114 | 78 (68.42) | 1.87 (1.32-2.65) |  |  | 116 | 81 (69.83) | 1.75 (1.26-2.43) |  |  | 230 | 159 (69.13) | 1.74 (1.38-2.21) |  |
| BCLC stage | |  |  |  |  |  |  |  |  |  |  |  |  |  |
| 0/A | 225 | 72 (32.00) | 1.00 | < 0.001 |  | 202 | 74 (36.63) | 1.00 | < 0.001 |  | 427 | 146 (34.19) | 1.00 | < 0.001 |
| B/C | 208 | 128 (61.54) | 2.02 (1.43-2.85) |  |  | 231 | 145 (62.77) | 1.90 (1.36-2.65) |  |  | 439 | 273 (62.19) | 1.98 (1.56-2.52) |  |
| a Obtained from multivariable Cox regression analysis with adjustment for age, sex, smoking status, drinking status, cirrhosis, cancer embolus, BCLC stage, and AFP level. | | | | | | | | | | | | | | |
| Abbreviation: OS, Overall Survival; HBV, Hepatitis B Virus; HCC, Hepatocellular Carcinoma; HR, Hazards Ratio; CI, Confidence Interval; AFP, Alpha-Fetoprotein; BCLC, Barcelona Clinic Liver Cancer Classification. | | | | | | | | | | | | | | |

| 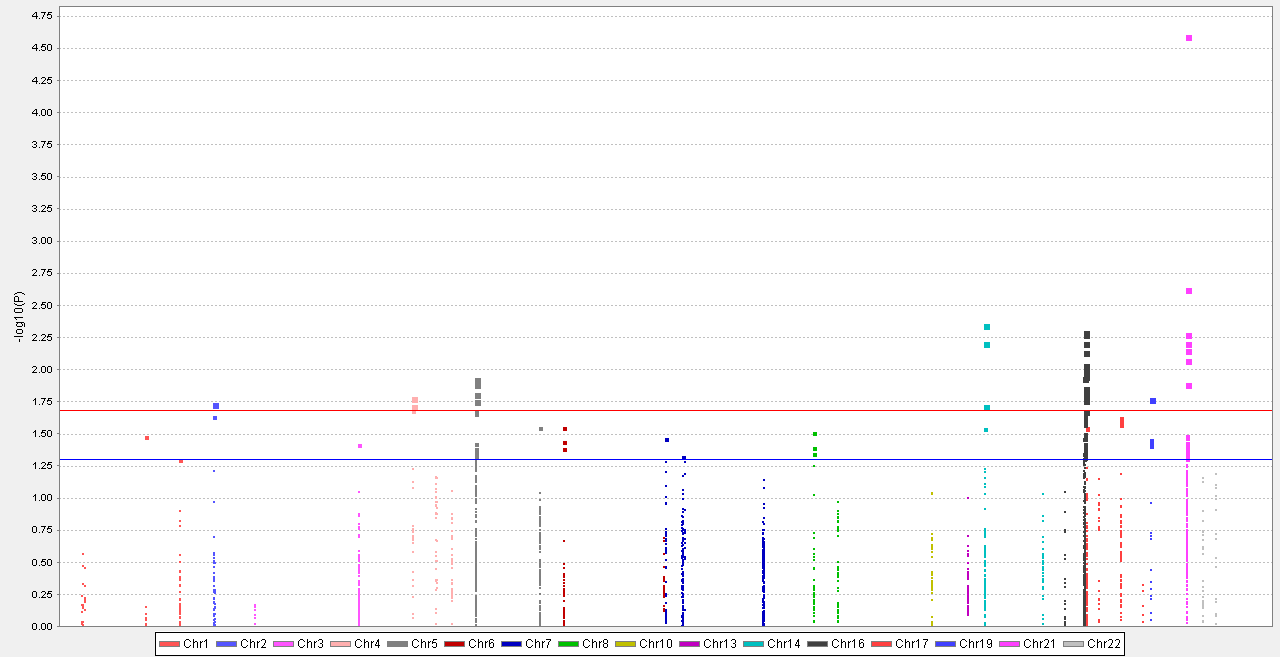 *ADARB1* rs1263790  METTL3 rs57884102 |
| --- |
| **Figure S1** Manhattan plot of the association of SNPs and HBV-HCC OS in the combined dataset.  Abbreviation: HBV, Hepatitis B Virus; HCC, Hepatocellular Carcinoma; OS, Overall Survival. |

| **A** | **B** |
| --- | --- |
| 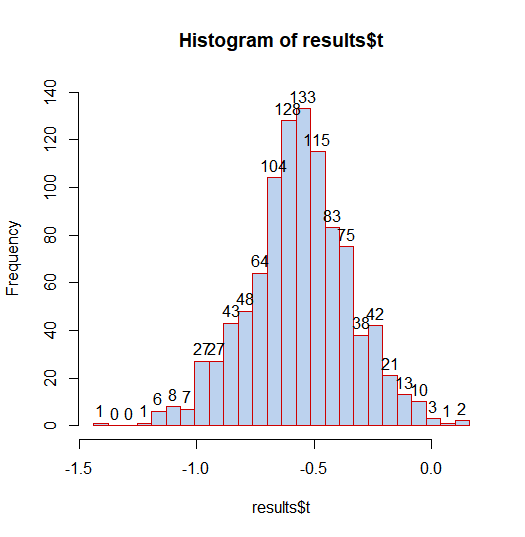 ***METTL3* rs1263790**  **95%CI of log_2_HR (-0.9965,-0.1204)** | 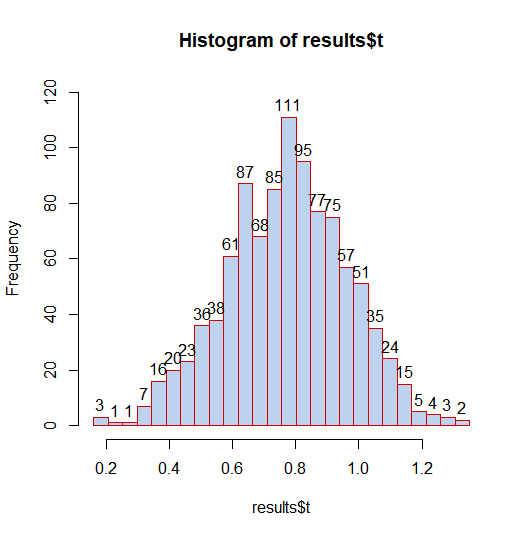 ***ADARB1* rs57884102**  **95%CI of log_2_HR (0.3620,1.1127)** |
| **Figure S2** The distribution histogram of 1000 HR values after grouped by bootstrapping for 1000 times. *METTL3* rs1263790 **(A)**, *ADARB1* rs57884102 **(B)**. | |

| **A** | **B** |
| --- | --- |
| 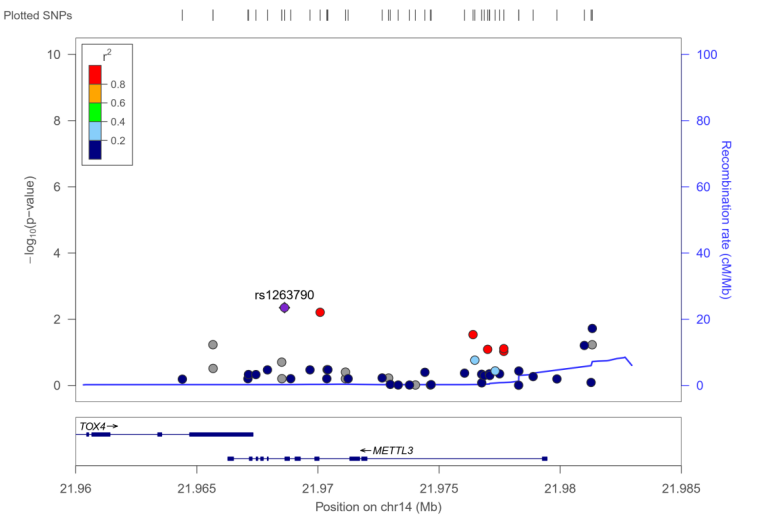 | 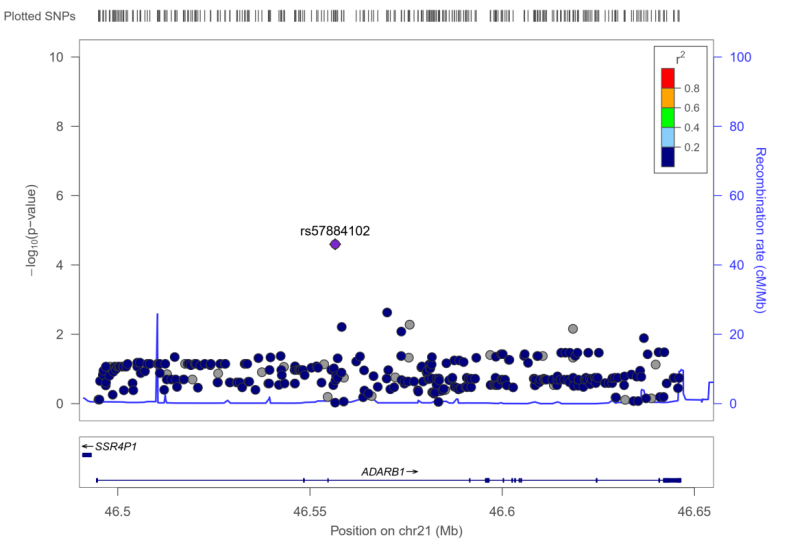 |
| **Figure S3** Regional association plots of the *METTL3* rs1263790 **(A)** and *ADARB1* rs57884102 **(B).** | |

| Multi-tissue eQTL Comparison  ENSG00000165819.11 METTL3 and chr14_21500477_G_A_b38(rs1263790) eQTL (Meta Analysis RE2 P-Value: 4.95564e-26) |
| --- |
| 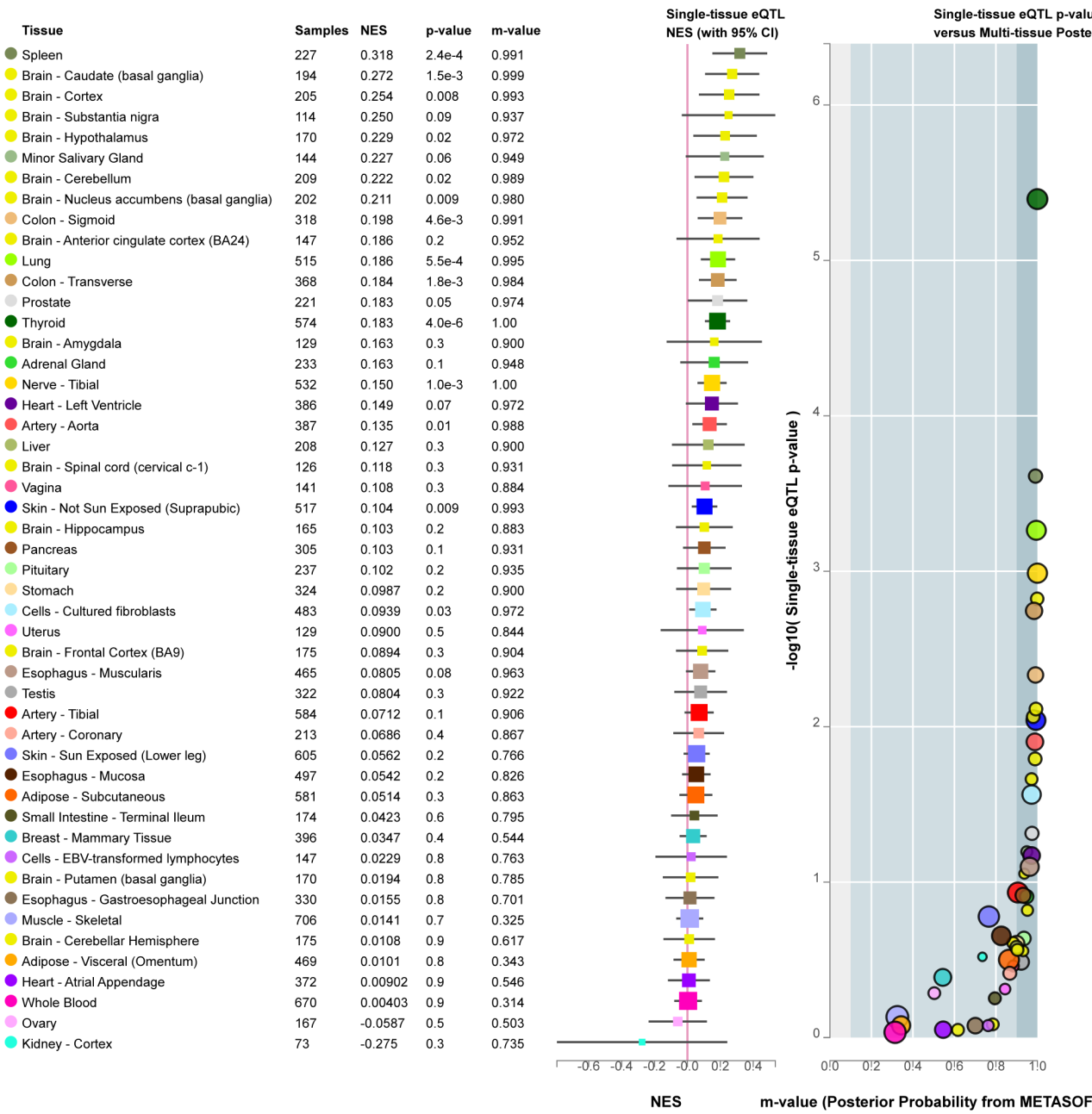 |
| **Figure S4** Multi-tissue eQTL analysis for *METTL3* rs1263790 in the GTEx database. |

| **A** | **B** |
| --- | --- |
| 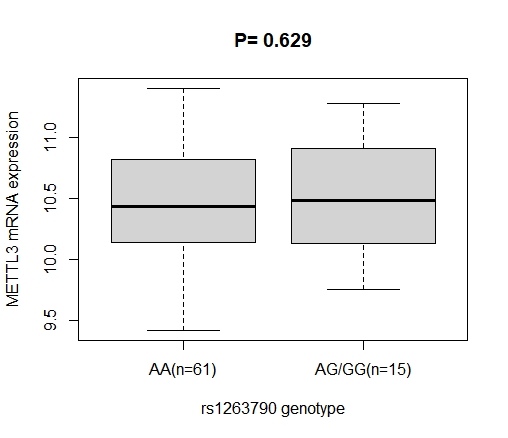 | 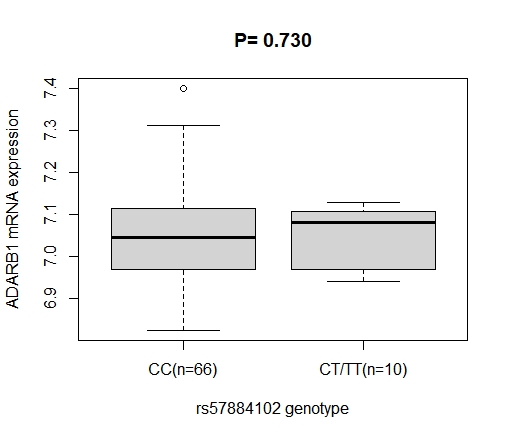 |
| **C** | **D** |
| 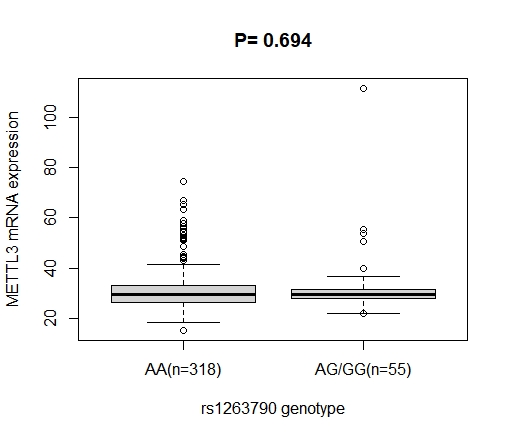 | 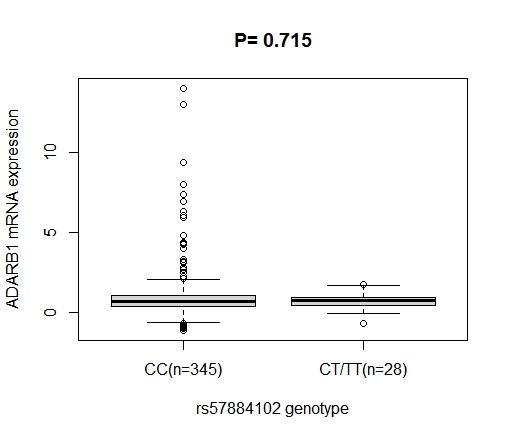 |
| **Figure S5** The expression quantitative trait loci (eQTL) analysis for *METTL3* rs1263790 and *ADARB1* rs57884102 from 1000 Genomes Project. Correlation of *METTL3* rs1263790 A > G **(A)** and *ADARB1* rs57884102 C > T **(B)** with mRNA expression in dominant genetic model in 76 Han Chinese in Beijing, China (CHB) populations. Correlation of *METTL3* rs1263790 A > G **(C)** and *ADARB1* rs57884102 C > T **(D)** with mRNA expression in dominant genetic model in 373 Europeans. | |
